# Novel Circular Rep-Encoding Single-Stranded DNA Viruses Detected in Treated Wastewater

**DOI:** 10.1128/MRA.00318-19

**Published:** 2019-05-02

**Authors:** Karyna Rosario, Christina M. Morrison, Kaitlin A. Mettel, Walter Q. Betancourt

**Affiliations:** a College of Marine Science, University of South Florida, Saint Petersburg, Florida, USA; b Department of Soil, Water and Environmental Science, University of Arizona, Tucson, Arizona, USA; DOE Joint Genome Institute

## Abstract

Here, we present the complete genome sequences of three circular replication-associated protein (Rep)-encoding single-stranded DNA (CRESS DNA) viruses detected in secondary treated and disinfected wastewater effluent. The discovered viruses, named wastewater CRESS DNA virus (WCDV)-1 to -3, represent novel viral species that seem to persist in wastewater effluent.

## ANNOUNCEMENT

Wastewater is a rich reservoir of viral diversity (1, 2). To safeguard public health, discovery efforts can exploit this diversity to identify novel indicators that can track virus removal efficiency through wastewater treatment. Here, we investigated treated wastewater effluent for the presence of potentially persistent viruses. Specifically, we searched for single-stranded DNA (ssDNA) viruses since these small viruses may be removed less effectively than other viral types through wastewater treatment (3, 4). We focused on ssDNA viruses encoding a replication-associated protein (Rep) within a circular genome, which include the smallest viruses known to infect eukaryotic organisms (5).

Effluent samples were collected from a single facility employing dissolved air flotation, four parallel five-stage Bardenpho processes, disc filtration, and chlorine disinfection for wastewater treatment. Virus recovery from 1 to 2 liters of effluent involved adsorption to negatively charged filter medium, followed by ultrafiltration using Centriprep YM-50 centrifugal filters (EMD Millipore) (6). DNA was extracted from ∼0.75 ml of retentate using the QIAamp UltraSens virus kit (Qiagen). Extracts were screened for circular Rep-encoding ssDNA (CRESS DNA) viruses through a degenerate PCR assay targeting the Rep of members of the *Circoviridae* (7), a group of vertebrate- and invertebrate-associated viruses (8). Degenerate PCR products were cloned and Sanger sequenced with vector primers. Sequences with BLAST matches to CRESS DNA viruses were used to design back-to-back primers to obtain complete genomes through inverse PCR. For this purpose, DNA extracts used for the degenerate PCR assay were amplified through rolling circle amplification (RCA) using the illustra TempliPhi kit (GE Healthcare). RCA products were used as the template for inverse PCR, and amplified genomes were cloned and sequenced using vector primers and primer walking using two additional primers. All cloning was performed using the CloneJET PCR cloning kit (Thermo Scientific).

Three CRESS DNA viruses, named wastewater CRESS DNA virus (WCDV)-1 to -3, were recovered from treated effluent (Table 1). Each genome was *de novo* assembled, with at least 3× coverage, from 10 to 16 reads in Geneious version R7 (Biomatters), using default parameters, and nonoverlapping open reading frames longer than 300 nucleotides were identified in SeqBuilder version 11.2.1 (Lasergene package; DNAStar). Similar to known CRESS DNA viruses (5), the WCDV genomes encode the Rep and a capsid protein within a small circular DNA molecule. The WCDVs also contain a putative origin of replication marked by a conserved nonamer (TAGTATTAC) at the apex of a predicted stem-loop structure (5). Based on the most conservative species demarcation threshold (77% genome-wide pairwise identity) for established CRESS DNA viral families (9), WCDV-1, WCDV-2, and WCDV-3 represent novel species (Table 1). Despite the use of a circovirus degenerate PCR assay, none of the WCDVs represent bona fide members of the *Circoviridae*. Notably, Rep- and capsid-coding sequences identified in WCDV genomes are arranged in the same strand, whereas *Circoviridae* members encode these proteins in the sense and antisense strands (8).

**TABLE 1:** Information about WCDV-1 to -3 genomes

Virus	Genome size (nt)^*a*^	Genome GC content (%)	Best BLAST match source (GenBank accession no.)^*b*^	Pairwise identity (%)^*c*^	Positive effluent samples (%) (n = 5)^*d*^	Detected after reverse osmosis^*e*^
WCDV-1	2,276	48.5	Pigs (MK142773)	51/57	80	No
WCDV-2	2,242	48.1	Shrews (KY370040)	46/56	100	Yes
WCDV-3	1,782	41.3	Wastewater (KY487913)	56/66	80	No

a nt, nucleotides.

b Best BLAST match based on BLASTx searches using the replication-associated protein (Rep)-coding sequence as a query.

c Rep amino acid/genome-wide sequence identities between WCDVs and their best BLAST match based on the Rep. See Table 2 from the study by Rosario et al. (11) for a summary of species demarcation criteria for established CRESS DNA viral families.

d Secondary treated and chlorinated effluent samples collected monthly between June and November 2018 were tested for WCDVs through PCR.

e Indicates if a given WCDV was detected by PCR in treated effluent after reverse osmosis.

Although we cannot predict the host for WCDVs, PCR assays indicate that these viruses are persistent in reclaimed effluent given that WCDVs were detected in samples collected over 3 months apart (Table 1). Moreover, WCDV-2 was detected after advanced treatment through reverse osmosis, which is considered an efficient treatment for virus removal (10). Future work will investigate how these previously undetected viruses perform as viral indicators of wastewater treatment efficiency for reuse applications.

### Data availability.

The genome sequences of WCDV-1 to WCDV-3 have been deposited in GenBank under the accession numbers MK583726, MK583727, and MK583728, respectively.
